# The Cyclic Antibacterial Peptide Enterocin AS-48: Isolation, Mode of Action, and Possible Food Applications

**DOI:** 10.3390/ijms151222706

**Published:** 2014-12-08

**Authors:** María José Grande Burgos, Rubén Pérez Pulido, María del Carmen López Aguayo, Antonio Gálvez, Rosario Lucas

**Affiliations:** Department of Health Sciences, University of Jaen, Campus Las Lagunillas s/n, 23071 Jaen, Spain; E-Mails: mjgrande@ujaen.es (M.J.G.B.); rppulido@ujaen.es (R.P.P.); mlaguayo@ujaen.es (M.C.L.A.); rlucas@ujaen.es (R.L.)

**Keywords:** enterocin, circular bacteriocin, antibacterial, membrane, food preservation

## Abstract

Enterocin AS-48 is a circular bacteriocin produced by *Enterococcus*. It contains a 70 amino acid-residue chain circularized by a head-to-tail peptide bond. The conformation of enterocin AS-48 is arranged into five alpha-helices with a compact globular structure. Enterocin AS-48 has a wide inhibitory spectrum on Gram-positive bacteria. Sensitivity of Gram-negative bacteria increases in combination with outer-membrane permeabilizing treatments. Eukaryotic cells are bacteriocin-resistant. This cationic peptide inserts into bacterial membranes and causes membrane permeabilization, leading ultimately to cell death. Microarray analysis revealed sets of up-regulated and down-regulated genes in *Bacillus cereus* cells treated with sublethal bacteriocin concentration. Enterocin AS-48 can be purified in two steps or prepared as lyophilized powder from cultures in whey-based substrates. The potential applications of enterocin AS-48 as a food biopreservative have been corroborated against foodborne pathogens and/or toxigenic bacteria (*Listeria monocytogenes*, *Bacillus cereus*, *Staphylococcus aureus*, *Escherichia coli*, *Salmonella enterica*) and spoilage bacteria (*Alicyclobacillus acidoterrestris*, *Bacillus* spp., *Paenibacillus* spp., *Geobacillus stearothermophilus*, *Brochothrix thermosphacta*, *Staphylococcus carnosus*, *Lactobacillus sakei* and other spoilage lactic acid bacteria). The efficacy of enterocin AS-48 in food systems increases greatly in combination with chemical preservatives, essential oils, phenolic compounds, and physico-chemical treatments such as sublethal heat, high-intensity pulsed-electric fields or high hydrostatic pressure.

## 1. Introduction

Bacteriocins can be defined as ribosomally synthesized antimicrobial peptides or proteins, which can be posttranslationally modified or not [1]. Bacteriocins can be classified in at least two major classes, which may include two or more subclasses each [2]. Class I comprises peptides that undergo extensive post-translational modification (exemplified by the lantibiotics nisin and lacticin 3147). Class II can include several subclasses, mainly class IIa (pediocin-like bacteriocins, such as pediocin PA-1/Ach and enterocin A), class IIb (two-peptide bacteriocins, e.g., enterocin L50 or plantaricins EF and JK), and class IIc (circular bacteriocins, such as enterocin AS-48). Circular bacteriocins are unique in that their *N*-terminal and *C*-terminal ends are linked by a peptide bond. The first circular bacteriocin characterized was enterocin AS-48 [3,4,5,6,7]. A total of 10 circular bacteriocins have been described up to date, which can be differentiated in two subgroups according to their sequence similarities and physico-chemical properties [8,9,10,11]. Subgroup 1 includes circular cationic peptides with a high (isoelectric point (pI) close to 10). This subgroup includes enterocin AS-48 from *Enterococcus faecalis*, garvicin ML from *Lactococcus garvieae* [12], uberolysin from *Streptococcus uberis* [13], carnocyclin A from *Carnobacterium maltaromaticum* [14], lactocyclin Q from *Leuconostoc mesenteroides* [15], leucocyclin Q from *Lactococcus* sp. [16], amylocyclin from *Bacillus amyloliquefaciens* [17], and circularin A from *Clostridium beijerinckii* [18]. The two circular bacteriocins included in subgroup 2 have much lower isoelectric points (pI 4–7): gassericin A from *Lactobacillus gasseri* [19,20], which is identical to reutericin 6 from *Lactobacillus reuteri* [20,21] and butyrivibriocin AR10 from *Butyrivibrio fibrisolvens* [22].

Bacteriocins can be found naturally in foods where the producer bacteria grow, e.g., in natural fermentations or even in contaminated and spoiled foods. A plethora of bacteriocins released by bacteria from foods have been characterized in the past decades, such as nisin, pediocins, lacticins, lactococcins, leuconocins, plantaricins, enterocins, carnobacteriocins, and others [23,24,25,26,27,28,29,30]. Many of these have been characterized at the biochemical and genetic level, and tested in food systems as biopreservatives against foodborne spoilage and pathogenic bacteria. As biopreservatives, bacteriocins should be used in combination with other preservation factors. The paradigm of bacteriocins is nisin, which is approved as a natural food preservative and widely used over the world.

## 2. The Bacteriocin Enterocin AS-48

Enterocin AS-48 is a circular bacteriocin produced by *Enterococcus faecalis* strains from both clinical sources [31,32] and from foods, mainly milk and traditional cheeses [33,34,35,36,37] including the food-grade strain *E. faecalis* UGRA10 isolated from a farmhouse raw sheep’s milk cheese [38]. A variant of enterocin AS-48 (enterocin AS-48RJ) differing in one amino acid residue was characterized in an *Enterococcus faecium* strain isolated from a home-made goat cheese [39]. Enterocin AS-48 can be recovered from liquid cultures of exponential as well as stationary-phase cells, indicating that it is produced during primary cell metabolism. A minimal medium was designed to facilitate high-yield bacteriocin production and rapid two-step purification based on cation exchange chromatography followed by semi-preparative reversed-phase high performance chromatography [3,40]. Enterocin AS-48 can also be produced on inexpensive food by-products such as whey permeate, which opens the way for an industrial-scale production of bacteriocin preparations suitable to be used as food additives [41]. Optimization of enterocin AS-48 production has been achieved by using a partially de-lactosed and de-mineralised derivative of whey, enriched in milk proteins (Esprion-300; DMV Int., Veghel, The Netherland). The critical factors for optimal enterocin AS-48 production in this medium were the pH stabilization at 6.55 and 1% glucose concentration [20]. Bacteriocin activity was expressed in arbitrary units (AU) against indicator strain *E. faecalis* S-47 (AU_S-47_). Under optimal fermentation conditions, up to 360 AU_S-47_/mL (which is equivalent to 104 µg of bacteriocin per mL) could be produced after 18 h cultivation. The levels of produced bacteriocin remained stable for up to 20 h, which is an additional advantage as it provides a broader margin for processing of cultured broths and bacteriocin recovery. The produced bacteriocin can be further processed by spray-drying, resulting in dry powder preparations suitable for food application [42].

**Figure 1:**
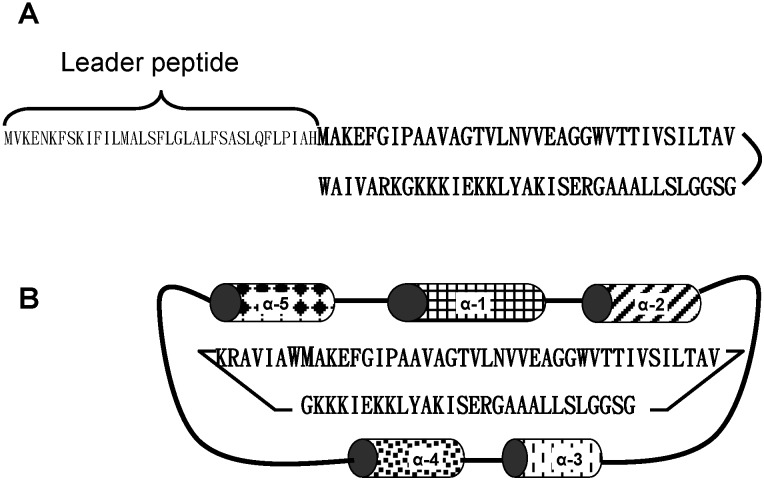
Processing of enterocin AS-48 structural gene product (**A**) by head-to-tail circularization; and α-helix arrangement of the mature circular peptide (**B**).

Composition analysis showed that enterocin AS-48 contains a high proportion of basic to acidic amino acids [3]. It also contained a high proportion (49%) of hydrophobic amino acids (Ala, Pro, Val, Met, Ile, Leu, and Phe) and uncharged hydrophilic amino acids (Ser, Gly, Thr, and Tyr). Enterocin AS-48 has 70 amino acid residues in total [4]. It does not contain modified amino acid residues or disulfide bridges. The mature molecule has a molecular mass of 7.14 kDa, and a pI of 10.09. Analysis of the genetic determinants for bacteriocin production indicated that enterocin AS-48 structural gene encodes for a 105-aminoacid prepeptide, which is further processed by removal of a 35-amino-acid signal peptide [5]. A peculiar feature of enterocin AS-48 is that its *N*- and *C*-terminal ends are linked by a peptide bond formed between the *N*-terminal methionine (Met^1^) to the *C*-terminal tryptophan (Trp^70^), yielding a circular structure [4] (Figure 1). At the time of its molecular characterization, it was the first circular bacteriocin described. Carnocyclin A from *Carnobacterium maltaromaticum* and uberolysin A from *Streptococcus uberis* are similar in structure to enterocin AS-48, even though they have low sequence identities [8].

The secondary and tertiary structure of enterocin AS-48 has been deciphered. The structure of enterocin AS-48 consists of a globular arrangement of five α-helices enclosing a compact hydrophobic core [43,44]. The head-to-tail union lies in the middle of helix 5, and has a pronounced effect on the stability of the three-dimensional structure of the molecule. Its amino acid sequence reveals a highly asymmetrical distribution of positive charges, since all 10 basic amino acid residues found in the molecule are contained in a segment of 26 residues out of a total of 70. Therefore, this region has the highest electrostatic potential. Circularity and compact folding confer enterocin AS-48 molecules a remarkable stability to extremes of pH, heat, and denaturing agents. Such stability is very convenient for application of the bacteriocin in food systems. Furthermore, enterocin AS-48 can be degraded by proteases of the intestinal tract (trypsin, pepsin), theoretically decreasing its possible impact on the gut microbiota for bacteriocin molecules ingested together with food. By contrast, it is resistant to carboxypeptidases and aminopeptidases. A nicked form and fragments derived from enterocin AS-48 by limited proteolysis with thermolysin still retained antibacterial activity against *Listeria monocytogenes*. However, the detected activity was between 300 and 1000 times lower compared to the intact peptide [45].

The genetic determinants of enterocin AS-48 are coded in a pheromone-responsive plasmid. The AS-48 gene cluster contains ten genes, including the bacteriocin structural gene, an immunity protein, and genes encoding two ATP-binding cassette (ABC) transporters [6]. The gene cluster for enterocin AS-48 production was cloned in several lactic acid bacteria (LAB) strains of the genera *Enterococcus*, *Lactococcu*s and *Lactobacillus* [46]. Both bacteriocin production and immunity were satisfactorily expressed in *Enterococcus* hosts. However, no bacteriocin production could be detected in lactococci or lactobacilli, although expression of a partial level of resistance against AS-48 was always detected. Fusion proteins of linear or permutated circular forms of enterocin AS-48 could be expressed in *Escherichia coli*, but they showed a strong tendency to aggregate. Hybrid fragments from fusion proteins recovered after non-specific cleavage with enterokinase and high-performance liquid chromatograpy (HPLC) repurification were shown to have anti-*Listeria* activity [47].

## 3. Inhibitory Spectrum of Enterocin AS-48

Enterocin AS-48 was shown to have antibacterial activity exclusively, with no activity against yeasts and molds or other eukaryotic cells being detected. Bacteriocin addition did not show any effect on intact cells of *Sacharomyces cerevisiae* or the amoebae *Naegleria fowleri* and *Acanthamoeba*. The bacteriocin concentration necessary to induce partial morphological changes (visible under a microscope) in a Vero cell line (100 μg/mL) was exceedingly higher than the inhibitory concentrations for most bacteria (between 1.5 and 10 μg/mL).

Most of the Gram-positive bacteria tested were highly sensitive to enterocin AS-48 [48]. Addition of low concentrations of enterocin AS-48 to exponential cultures of all these strains resulted in the immediate cessation of growth and also in a steady decrease in cell viability. Additionally, loss of turbidity was also detected in many cases immediately or following a variable lag period after enterocin AS-48 addition, indicating a bacteriolytic mode of action. Bacteria containing mycolic acids in their cell walls (*Corynebacterium*, *Mycobacterium* and *Nocardia*) were highly sensitive to enterocin AS-48. However none of them showed bacteriolysis after treatment with bacteriocin. Other Gram-positive bacteria such as *Micrococcus* and *Staphylococcus* species were less sensitive, and none of them were lysed after bacteriocin addition. Species of the genus *Listeria* were also sensitive to enterocin AS-48. *L. monocytogenes* could be inhibited in broth by a bacteriocin concentration as low as 0.1 µg/mL. *Brochothrix thermosphacta* and lactic acid bacteria (belonging to genera *Lactobacillus*, *Lactococcus*, *Leuconostoc* and *Pediococcus*) also were bacteriocin-sensitive. Both aerobic or facultatively anaerobic endospore formers (*Bacillus cereus*, *Bacillus coagulans*, *Bacillus subtilis*, *Paenibacillus* spp., *Bacillus licheniformis*, *Bacillus macroides*, *Alicyclobacillus acidoterrestris*, *Alicyclobacillus acidocaldarius*, *Geobacillus stearothermophilus*) as well as anaerobic endospore formers (*Clostridium perfringens*¸ *Clostridium sporogenes* and *Clostridium tetani*) were bacteriocin-sensitive.

Early studies showed that many species of Gram-negative bacteria were also inhibited by enterocin AS-48 [48]. However, Gram-negative strains were about ten times less sensitive to enterocin AS-48 than Gram-positive bacteria when tested in solid medium, presumably due to the protective effect of bacterial outer membrane. The most sensitive species were *Myxococcus*, *E. coli* and *Rhizobium* strains. *Myxococcus* strains were the only Gram-negative bacteria that lysed after addition of bacteriocin concentrations similar to those used for inhibiting Gram-positive bacteria. Other Gram-negative bacteria were less sensitive, such as *Agrobacterium*, *Salmonella*, *Shigella*, *Pseudomonas* and *Klebsiella.* All of them required high concentrations for inhibition (above 100 µg/mL), and none of them was lysed. Further studies have confirmed the higher tolerance of Gram-negative bacteria to enterocin AS-48, and how the application of treatments that destabilized the bacterial outer membrane dramatically increase bacteriocin sensitivity. Combined treatments of enterocin AS-48 with sublethal heat, chelators (such as ethylenediaminetetraacetic acid (EDTA) or tripolyphosphate), polymyxin B, or with pulsed electric fields of high intensity (HIPEF) improve considerably the inactivation of *E. coli* and *Salmonella enterica* both in culture media and in foods [49,50,51,52].

Bacterial endospores are highly resistant to environmental factors, such as heat, UV, radiation, pulsed electric fields, high hydrostatic pressure, chemical antimicrobials, and bacteriocins. They are therefore of great concern in the food industry, since endospore formers may cause food poisoning or food spoilage. When endospores of the food poisoning bacterium *Bacillus cereus* were investigated for sensitivity to enterocin AS-48, it was found that the viability of *B. cereus* dormant endospores was not affected by incubation with enterocin AS-48 (50 µg/mL) for 3 h, indicating that intact endospores were highly resistant to the bacteriocin [53]. Furthermore, release of dipicolinic acid was not inhibited in the presence of bacteriocin in *B. cereus* spores induced to germinate, indicating that initiation of germination was not affected by enterocin AS-48. Nevertheless, inactivation by enterocin AS-48 could be detected as early as 10 min after induction of germination at 37 °C, as shown by the marked reductions of viable cell counts obtained for bacteriocin concentrations of 25 to 50 µg/mL. During prolonged incubation (90–120 min), bacteriocin sensitivity increased, although there was a small fraction of superdormant spores that were not inactivated. Superdormant spores are usually a small fraction of the endospore population, but they germinate extremely slowly [54] and often cause problems in the food industry. Conditions inhibiting endospore germination such as incubation at 5 °C for 2 h precluded inactivation by the added bacteriocin. However, when cold-stored endospores were transferred to 37 °C and engaged in germination and outgrowth, the added bacteriocin significantly reduced viable cell counts. This observation could have implications for biopreservation of cold-stored samples accidentally exposed to temperature abuse.

Since bacterial endospores may survive conventional heat treatments applied to foods and at the same time heat treatments may activate endospore germination, it would be interesting to investigate the combined effect of enterocin AS-48 and heat. When *B. cereus* endospores (not induced to germinate) inoculated in rice foods were heat treated, it was observed that survivor viable counts decreased considerably if heat treatments were applied in combination with 16 µg/mL enterocin AS-48 [55]. The combined effect of enterocin AS-48 and heat treatments was corroborated on *Bacillus licheniformis*. Endospores from this bacterium may require the application of high-intensity heat treatments for inactivation. When *B. licheniformis* endospores inoculated in a commercial apple cider were heat-treated in the presence of enterocin AS-48, viable cell counts decreased in proportion to the heat treatment (85 to 95 °C for 1 to 6 min) and the added bacteriocin concentration, detecting no survivors after treatments at 95 °C for 4 min and 6 µg/mL enterocin AS-48 or 1 min and 12 µg/mL enterocin AS-48 [56]. Calculated thermal death *D* value (the time in minutes at a given temperature required to inactivate one log cycle of the target microorganism) and *z* value (the temperature change required to change the *D* value by a factor of 10) were also significantly reduced in combination with the bacteriocin. Since heat treatments activate endospore germination, it is tempting to suggest that, following germination activation by heat, the microorganism was then killed by the presence of bacteriocin.

Inactivation of endospores was also investigated in the fruit juice spoilage bacterium *Alicyclobacillus acidoterrestris.* Endospores of this bacterium were found to be extremely sensitive to enterocin AS-48 even without thermal treatment [57]. After one-min contact in combination with 2.5 µg/mL bacteriocin, no viable cells could be recovered from endospore suspensions of about 6 log units. Results from electron microscopy of the treated endospores revealed substantial damage in endospore structure. The short contact-time with bacteriocin required to obtain the observed effects suggested that the bacteriocin adsorbed rapidly to bacterial endospores. This rapid adsorption could be explained in terms of endospore structure and also because of the low pH of the growth medium and juice used for the assays. Since the net positive charge of enterocin AS-48 increased as the pH decreased, a stronger interaction with negatively-charged endospore surface groups would be expected at lower pH. A very rapid adsorption of bacteriocin to endospores was also shown for *Geobacillus stearothermophilus*, which was sensitive to a bacteriocin concentration as low as 1.75 µg/mL [58]. When endospores treated with bacteriocin were then treated with trypsin, adsorbed bacteriocin could be proteolytically inactivated and its biocidal effects counteracted.

## 4. Bacteriocin Mode of Action

Early kinetic studies carried out on *E. faecalis* S-47 sensitive cells indicated a multi-hit kinetic mechanism of action (which means that more than one bacteriocin molecule is required to inactivate one bacterial cell), requiring a very low number of not more than 10 enterocin AS-48 molecules to render this bacterium nonviable [59]. However, shortly after bacteriocin addition, the bactericidal action could be neutralized by bovine heart cardiolipin or trypsin (indicating a reversible early stage interaction), but this was not possible after prolonged incubation with enterocin AS-48. These observations were consistent with the short interval of time between bacteriocin addition and the detection of biological effects (e.g., alteration of ion permeability, precursors uptake and biosynthesis as we will see further on). These early effects would lead to a rapid loss of cell viability, thus making cell rescue unlikely after prolonged incubation.

Enterocin AS-48 also exerts a bactericidal, but not bacteriolytic mode of action on *E. coli* K-12 [60]. The effect of different bacteriocin concentrations on growth and viability of *E. coli* K-12 is similar to that determined in *E. faecalis* S-47. However, the bacteriocin concentrations required to reduce the number of viable cells significantly (150 μg/mL) were much higher than those used for inhibition of Gram-positive bacteria (such as *Enterococcus* and *Bacillus* species). An atypical sigmoid curve was obtained for the relationship between bacteriocin dose and percentage of surviving cells after incubation for 5 min with increasing bacteriocin concentrations. Therefore, three different responses were obtained depending on bacteriocin dose: First, concentrations below 10 AU against *E. coli* K-12 per mL (AU_K-12_/mL) induced a negligible decrease in the surviving fraction, suggesting a multi-hit kinetics mechanism of action. In the second place (10–100 AU_K-12_/mL) the decrease in the percentage of viable cells was directly proportional to increments in bacteriocin concentrations. And finally, bacteriocin concentrations above 200 AU_K-12_/mL had very little effect on cell viability (saturation).

Further studies were carried out on the biological activity of enterocin AS-48 by measuring its effects on several cellular parameters. When *E. faecalis* was used as sensitive strain [59], bacteriocin addition (8 AU_S-47_/mL or about 2.3 µg/mL) impaired the capacity of intact cells to accumulate radiolabeled precursors [6-^3^H]thymidine, [5,6-^3^H]uridine, l-[4,5-^3^H]leucine and [1-^14^C]acetate and to incorporate them into the respective macromolecules (protein, RNA, DNA and cell wall peptidoglycan). Incorporation of all precursors dropped below 40% of the initial incorporation within 1 min after bacteriocin addition, and it was significantly reduced (below 10%) after 5 min of incubation. Also, the capacity to maintain the cytoplasmic levels of K^+^ was completely lost 5 min after bacteriocin addition, concomitantly with an increase in the cellular Na^+^ content. Furthermore, addition of enterocin AS-48 markedly inhibited the capacity for O_2_ uptake within 30 s after bacteriocin addition, while control cultures continued active consumption over the entire period monitored. Altogether, these results strongly suggested that the cytoplasmic membrane becomes damaged by bacteriocin.

Similar studies were also conducted on *E. coli* K-12 using the same precursors for protein, RNA and DNA synthesis, and d,l-(+)-*meso*-diamino-[G-^3^H]pimelic acid for cell wall biosynthesis and a much higher bacteriocin concentration (100 AU_K-12_/mL or *ca.* 134 µg/mL) [60]. In this bacterium, the main physiological effects observed following enterocin AS-48 addition consisted of a gradual (but not drastic) cessation in the incorporation of radiolabeled precursors into macromolecules as well as in the rate of uptake of labeled precursors. The rate of O_2_ consumption also decreased gradually. This simultaneous slowdown of all metabolic pathways occurred in parallel to the cessation of growth as estimated from optical density.

In order to gain more insights into the mechanism of action of enterocin AS-48, the effects of this bacteriocin on retention of radiolabeled rubidium (^86^Rb^+^) or to accumulate external ^86^Rb^+^ as an analog of K^+^ were also studied, since this method leads to a more accurate determination in shorter times. Addition of enterocin AS-48 to *E. faecalis* cells loaded with ^86^Rb^+^ induced a rapid efflux of this ion as well as an immediate failure to accumulate it from the medium [61]. Similar ^86^Rb^+^ efflux was observed in other Gram-positive bacteria assayed (*Corynebacterium glutamicum* or *Bacillus subtilis*), since these bacteria lost 92% and 83% of the accumulated rubidium, respectively, after 5 min of incubation with enterocin AS-48 [61].

The effects of enterocin AS-48 on the membrane electrical potential of *E. faecalis* S-47 cells were measured both at rest and in cells energized with glucose [61]. Addition of enterocin AS-48 led to a rapid decrease in the membrane potential, comparable to the effect obtained for the ATPase inhibitor *N*,*N*’-dicyclohexylcarbodiimide used as control. No difference were found in the action of enterocin AS-48 on cells at rest or energized, although the membrane potential was slightly higher in the latter. Consequently, abolition of the membrane potential by enterocin AS-48 would rapidly impair transport of amino acids and other precursors, resulting in the observed inhibition of the biosynthetic pathways in enterocin AS-48 treated cells [61].

Experiments carried out on transport and permeability in membrane vesicles derived from *E. faecalis* and *E. coli* indicated that both types of vesicles were highly sensitive to enterocin AS-48 [61]. Vesicles from *E. coli* failed to accumulate proline if they were treated with enterocin AS-48 before addition of the energizing electron pair Ascorbate/Phenazinemethosulfate, indicating that no membrane potential was required for enterocin AS-48 to act. Although the removal of the cell wall did not diminish the sensitivity of *E. faecalis*, the vesicles obtained from *E. coli* were far more sensitive to enterocin AS-48 than intact cells, in which the effect of enterocin AS-48 were observed at concentrations 10 to 15 times higher. These results corroborate the previously suggested protective role of the bacterial outer membrane against enterocin AS-48 in Gram-negative bacteria. The experiments carried with membrane vesicles also confirmed that enterocin AS-48 does not require a preexisting membrane potential for interaction with bacterial membranes. This idea was corroborated by studies on the effects of enterocin AS-48 on artificial lipid systems, such as liposomes and planar phospholipid bilayers. Liposomes constructed from asolectin were used as an artificial membrane system to measure the effects of enterocin AS-48 [61]. Addition of low bacteriocin concentration (5 μg/mL) leads to the free diffusion of low-molecular weight solutes (such as radiolabeled uridine or rubidium), while those solutes of a higher mass, such as radiolabeled dextran, were retained or diffuse much more slowly. However, it was also observed that both prolonged incubation and an increase in bacteriocin concentration caused a more chaotic membrane disorganization. Untreated liposomes appeared under electron microscopy as unilamellar structures of variable size, but after being treated with enterocin AS-48 for 15 min, they appeared as multilamellar aggregates. Intermediate stages in which very small vesicles or blebs seemed to protrude from or to adhere to larger liposome structures were also observed.

**Figure 2:**
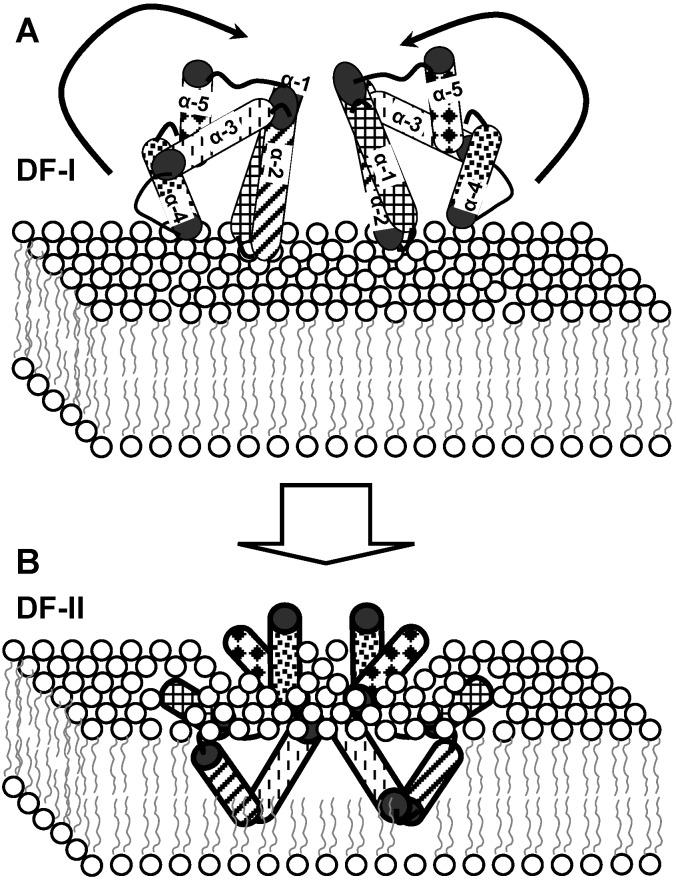
Proposed mechanism for transition of enterocin AS-48 from water-soluble DF-I (**A**) to the membrane-bound DF-II stage (**B**).

Evidence for the intimate mode of action of enterocin AS-48 was obtained from conductance experiments carried out on planar phospholipid bilayers. The electric resistance of planar bilayers made of asolectin was disturbed by addition of low bacteriocin concentrations [61]. Conductance measurements revealed that, after a delay period, addition of enterocin AS-48 induced a rapid accumulation of electrical events corresponding to the opening of channels of a recorded conductance of 12 to 18 picosiemens (pS). Assuming a membrane thickness of 6 nm, the average pore diameter formed by enterocin AS-48 could be estimated roughly in 0.7 nm [61]. The formation of ion channels or pores of low specificity by the insertion of enterocin AS-48 molecules into the cytoplasmic membrane could provide an efficient mechanism for the induction of cell depolarization, allowing the diffusion of low-molecular-weight solutes from the cells, dissipating the membrane potential and rendering the cells nonviable. Interestingly, carnocyclin A has also been shown to permeabilize liposomes and/or lipid bilayers [62]. Enterocin AS-48 tends to form aggregates and dimers in aqueous solutions. Based on the crystal structure of enterocin AS-48 [63], the proposed mechanism of action suggests that the two different stages of molecular dimer association, dimer form I (DF-I) and DF-II, are involved in changing from the water-soluble DF-I to the membrane-bound DF-II stage at the membrane surface (Figure 2). This transition implies a 90° rotation of each protomer within DF-I, in a way that the partially hidden hydrophobic helices H1 and H2 become solvent accessible [63]. This would permit enterocin AS-48 molecules to insert into the bacterial cytoplasmic membrane. The model for DF-I state is compatible with direct interaction with membrane phospholipids [63]. In the DF-I state, a belt of positively charged amino acid residues and glutamic side-chains (particularly Glu58) could interact with the phosphate polar heads of phospholipids. In addition, the aromatic Tyr54 and Trp70 residues, with affinity for the lipid-water interface, could interact with phospholipid ester bond carbonyl groups as they are located below the plane formed by glutamic side-chains. These results, together with the observed effects on asolectin liposomes and lipid bilayers would suggest that enterocin AS-48 can interact with bacterial membranes without the need of a receptor. An increasing number of studies indicate that several other bacteriocins interact with specific bacterial cell receptors of different kinds, such as lipid II and related cell wall precursors, the mannose phosphotransferase system, a Zn-dependent metallopeptidase, undecaprenyl pyrophosphate phosphatase, or the maltose ABC transporter [64]. The last one is used as receptor by the circular bacteriocin garvicin ML. Since many of the circular bacteriocins are thought to share a common structural motif, the possibility that they may share a cellular receptor cannot be ruled out. It has been argued that circular bacteriocins may have a receptor-independent, non-specific effect at high concentrations and a receptor-dependent, specific activity at low concentration [8]. Nevertheless, interaction of enterocin AS-48 with asolectin liposomes and lipid bilayers (which is presumed to be receptor-independent) was observed at bacteriocin concentrations (between 1 and 4 µg/mL) even lower than those required to see an effect on intact cells (10 µg/mL) [61].

In addition to its bactericidal action, enterocin AS-48 exerts a bacteriolytic effect on many of the bacteria tested [48]. This seems to be secondary to the primary membrane action. Some other membrane-damaging peptides (such as nisin and pediocin PA-1/AcH) can also induce bacterial cell autolysis [65,66]. It has been suggested that, while the cell membrane is the primary target, membrane depolarization results in loss of control over the cell wall lytic enzymes. Induction of autolysis by enterocin AS-48 seems to be dependent on cell biosynthetic activity at the moment of bacteriocin addition, since inhibition of protein or RNA synthesis before incubation with enterocin AS-48 results in a marked decrease in bacterial autolysis. These results are in accordance with the role of protein synthesis in autolysin activation. Autolysins are considered to be synthesized in the cytoplasm as inactive precursors (proautolysins), which are activated by a native proteinase (activator) during or more likely after transport through the cytoplasmic membrane to wall synthesis sites [67]. Autolysis by enterocin AS-48 is also dependent of temperature, requires a basal membrane potential, can be inhibited by EDTA and cardiolipin and activated by trypsin (a known autolysin activator in altered cell walls of enterococci) [48]. Induction of autolysis by bacteriocins could be of technological advantage in food processing, such as in cheese ripening, contributing to a faster release of intracellular enzymes responsible for flavor development.

One interesting question is if and how cells respond to the presence of bacteriocins. In order to gain insight into this issue, the effect of enterocin AS-48 challenges on vegetative cells of *Bacillus cereus* ATCC 14579 was investigated by transcriptome analysis [68]. Of the 5200 genes analysed, expression of 24 genes was found to change significantly after a 30 min treatment with a subinhibitory bacteriocin concentration of 0.5 μg/mL. One operon involved in arginine metabolism was significantly down-regulated, together with genes for carbamate kinase, arginine deiminase and ornithine carbamoyltransferase. Most of the up-regulated genes encode membrane-associated or secreted proteins with putative transmembrane segments or signal sequences, respectively. Some of them were related with energy metabolism, and it seems likely that they were overexpressed to compensate for interference of AS-48 with energy generation. Others, like the antiholin-like protein could possibly interfere with AS-48 interaction with the bacterial cytoplasmic membrane. The BC4206-BC4207 operon was found to be the most up-regulated target. BC4206 codes for a PadR type transcriptional regulator, while BC4207 codes for a hypothetical membrane protein. Using real-time qPCR, it was shown that these genes are up-regulated when cells are treated with enterocin AS-48, but not upon nisin treatment. Upon overexpression of BC4207 in *B. cereus*, we observed an increased resistance against enterocin AS-48, with slight changes in the minimum inhibitory concentration (MIC) from 2.5 to 4.5 μg/mL. Expression of BC4207 in *B. subtilis* 168, which lacks this operon also showed increased resistance against enterocin AS-48 (with a MIC increase from 1 to 5 μg/mL).

## 5. Antibacterial Effects of Enterocin AS-48 in Food Systems

The potential of enterocin AS-48 to control foodborne pathogens has been demonstrated in several food products of animal origin, including meats, dairy products, seafood, as well as many different types of vegetable-based foods [69,70] (Table 1).

In a meat sausage model system with added enterocin AS-48, no viable *L. monocytogenes* were detected after 6 and 9 days of incubation at 20 °C [71]. When the bacteriocin-producing strain *E. faecalis* A-48-32 was used as inoculum at about 10^7^ CFU/g, *Listeria* counts decreased progressively from the start of the experiment, being below detection level at day 9. In the same meat system, enterocin AS-48 inhibited proliferation of *S. aureus* when added at concentrations of 30 or 40 µg/g, achieving significant reductions in viable cell counts of 2 and 5.3 log units, respectively, compared to the untreated controls [72]. Inoculation with the bacteriocin-producing strain (about 10^7^ CFU/g) also provided satisfactory results, producing bacteriocin in the meat sausage and reducing the viable cell counts of staphylococci down to 1.06 log CFU/g by day 9. In a cooked ham model system, enterocin AS-48 (20, 40 and 60 µg/g) alone was active against *L. monocytogenes* in samples stored at 5 and 15 °C, but it was not sufficient to avoid regrowth of *Listeria* during the 60 days storage period [73]. The effect on *S. aureus* was much more limited, even in samples stored at 5 °C. The combinations of enterocin AS-48 and chemical preservatives (nitrite/nitrate, pentasodium tripolyphosphate, sodium benzoate or potassium sorbate) improved the anti-listeria effect during refrigeration storage. The combination of nitrate/nitrite (0.007%) and enterocin AS-48 (40 µg/g) greatly enhanced the inhibitory effect against both bacteria. This effect was especially significant for *L. monocytogenes*, whose counts remained below detection levels during the cold storage period. Treatment with 60 µg/g enterocin AS-48 in combination with 0.15% sodium pyrophosphate provided the best results against both bacteria, reducing *Listeria* counts below detectable levels from day 1 of storage and keeping staphylococcal counts below 1 log CFU/g from day 15 on. In fuet (a low acid fermented sausage), the effect of enterocin AS-48 on *L. monocytogenes*, *S. enterica*, and *S. aureus* was investigated during ripening and storage at 7 °C or at room temperature in samples treated or not by high hydrostatic pressure [74]. Added enterocin AS-48 caused a rapid and drastic decrease in *L. monocytogenes* and a significant inhibition for *Salmonella* at the end of ripening (day 10). Fuets pressurized at 400 MPa in combination with enterocin AS-48 had lower counts of *Salmonella* compared to the single pressure treatment. Added bacteriocin did not significantly enhance inactivation of staphylococci by HHP treatment in fuets. Inhibition of meat spoilage bacteria could also be accomplished by enterocin AS-48, as shown by studies carried out in cooked ham with *Lactobacillus sakei*, *Brochothrix thermosphacta*, and *Staphylococcus carnosus* [75]. Although *L. sakei* was completely inactivated by enterocin AS-48 at 60 µg/g, the bacteriocin was most effective when used in combination with chemical preservatives. Combinations of enterocin AS-48 at 40 µg/g with nitrate/nitrite, pentasodium tripolyphosphate, sodium pyrophosphate, sodium acetate, and sodium lactate reduced *L. sakei* below detection levels from the beginning to end of storage. Enterocin AS-48 (40 µg/g) was also active against *B. thermosphacta* and *S. carnosus*, reducing both bacteria by more than 3 log cycles.

The bacteriocin-producer strain *E. faecalis* A-48-32 shows a strong capacity for growth in milk and to produce enterocin AS-48 [76]. In cocultures with *B. cereus* carried out in skim milk, the bacteriocin producer was able to completely inactivate the bacilli after 72 h incubation at 30 °C. Enterotoxin production was also inhibited. In cheese, *E. faecalis* A-48-32 was also able to produce enterocin AS-48 [76]. Growth of cheese starter cultures and lactic acid production were not affected by addition of this bacteriocin-producing strain. During ripening of non-fat cheese challenged with *B. cereus*, the produced bacteriocin was stable during the 90 days ripening period, and it was also able to reduce the population of bacilli in cheeses. The recovered bacilli population was composed mainly of endospores, indicating that vegetative cells were inactivated by the produced bacteriocin. Another study addressed the effect of enterocin AS-48 against *S. aureus* in skim milk and in fresh cheese [77]. In skim milk, added enterocin AS-48 inhibited *S. aureus* in a concentration-dependent mode. The efficacy of bacteriocin improved considerably in combination with a sublethal heat treatment. In cocultures carried out in skim milk at 28 °C, the enterocin AS-48 producer strain was also able to control staphylococci, in a way that depended on the initial enterococci-to-staphylococci ratio. In cocultures carried out in cheeses, the produced bacteriocin had strong anti-staphylococcal activity, and kept staphylococcal populations at least 1 log CFU/g below controls throughout storage for at least 28 days at 4 °C. In a further study, a bacteriocin preparation obtained by spray drying was shown to achieve partial inhibition of *S. aureus* or complete inactivation of *L. monocytogenes* inoculated in skim milk [42]. The anti-staphylococcal activity of enterocin AS-48 in milk has also been investigated in combination with high-intensity pulsed-electric field (HIPEF) treatments [78]. Synergistic effects were detected for combinations of enterocin AS-48 and nisin, enterocin AS-48 and HIPEF, and enterocin AS-48 plus nisin and HIPEF. The combination of the two bacteriocins plus HIPEF (800 μs) achieved over 6 log reductions in viable cell counts.

Enterocin AS-48 has not been investigated very much in seafood products. Nevertheless, dipping sardine fillets in a bacteriocin solution was reported to have low effect on the overall microbial load, except for some reduction in histamine- and tyramine-forming lactic acid bacteria [79]. Consistently, the levels of different biogenic amines after storage were significantly reduced by several fold in the bacteriocin-treated samples.

Foods of vegetable origin have been investigated in detail as targets for application of enterocin AS-48 as a biopreservative. Application of washing treatments with enterocin AS-48 alone or in combination with other antimicrobials was effective in the inactivation of *L. monocytogenes*, *B. cereus* and enteric bacteria in sprouts, as well as *L. monocytogenes* in whole fruit pieces and sliced fruits (strawberries, raspberries, blackberries, sliced melon, watermelon, pear, kiwi) [80,81,82,83]. The applied treatments could help to reduce transmission of foodborne pathogens through these raw foods, which have been implicated in foodborne outbreaks [84].

The combination of enterocin AS-48 with other antibacterial treatment was also effective in the inactivation of *L. monocytogenes* and *S. enterica* in ready-to-eat salads [85,86] and in the inactivation of *S. aureus* in different types of sauces [87]. In salads, inactivation of *L. monocytogenes* was potentiated by adding various essential oils and bioactive components from essential oils and plant extracts, while inactivation of *S. enterica* was significantly potentiated when the bacteriocin was added in combination with *p-*hydroxybenzoic acid methyl ester and with 2-nitropropanol. Anti-staphylococcal activity in sauces was potentiated significantly by the combined addition of enterocin AS-48 and phenolic compounds.

Enterocin AS-48 seems a good candidate for application in biopreservation of fruit juices. Addition of low enterocin AS-48 concentrations in juices artificially contaminated with vegetative cells as well as with endospores of *A. acidoterrestris* caused complete bacterial inactivation and afforded protection for up to 14 days in freshly made orange and apple juices, and for up to 60 to 90 days in several commercial fruit juices under storage temperatures in the range of 4 to 37 °C [57]. The thermophilic sporeformer *G. stearothermophilus* could also be inactivated rapidly by enterocin AS-48 in coconut milk and coconut water by a low bacteriocin concentration of 1.75 μg/mL [58].

Enterocin AS-48 has also been tested with satisfactory results against bacteria causing ropiness and other alterations in apple juice and apple cider, including the rope-forming strain *Bacillus licheniformis* LMG 19409 isolated from spoiled Normand ciders [56], exopolysaccharide producing lactic acid bacteria strains of *Lactobacillus collinoides*, *Lactobacillus diolivorans* and *Pediococcus parvulus* as well as 3-hydroxypropionaldehyde-producing *L. collinoides* strains isolated from spoiled apple ciders [88]. Activity against lactobacilli and pediococci in apple cider was potentiated by HIPEF treatments [89,90]. Inactivation of enteric pathogens in apple juice was also enhanced by bacteriocin addition, as in the case of *S. enterica* in combination with HIPEF [52] or *E. coli* O157:H7 in combination with EDTA and/or sublethal heat [50]. For *S. enterica*, the maximum inactivation (4.5-log cycles) was achieved with HIPEF treatment for 1000 μs in combination with 60 μg/mL of enterocin AS-48 and a treatment temperature of 40 °C. In energy drinks, the bacteriocin was effective in the inactivation of *L. monocytogenes* (1 μg/mL), *B. licheniformis* (12.5 μg/mL) and *S. aureus* (25 μg/mL) during storage at 37 °C and could be employed as a barrier in drinks with a less acidic pH [91].

In ready-to-eat rice-based foods (including boiled rice and in a commercial infant rice-based gruel dissolved in whole milk), bacteriocin addition (20–35 μg/mL) caused complete bacterial inactivation of psychrotrophic enterotoxigenic strains of *B. cereus*, both in samples stored in a temperature range of 6 to 37 °C and it also avoided enterotoxin production [55]. Commercial soups and purees supplemented with enterocin AS-48 were challenged with aerobic mesophilic endospore-forming bacteria. *B. cereus* was completely inhibited in all six vegetable foods tested (natural vegetable cream, asparagus cream, traditional soup, homemade style traditional soup, vegetable soup, and vichyssoise) by added enterocin AS-48 (10 μg/mL) for up to 30 days at 6, 15 and 22 °C [92]. Cocktails of strains composed of *B. cereus*, *B. macroides* and *Paenibacillus* sp., *Paenibacillus polymyxa*, and *Paenibacillus amylolyticus* showed higher bacteriocin resistance, requiring up to 50 μg/mL bacteriocin for complete inactivation in natural vegetable cream stored at 22 °C. Bactericidal activity against a cocktail of strains was greatly enhanced by phenolic compounds.

Incorporation of enterocin AS-48 (6 μg/mL) in low-acid vegetable canned foods (tomato paste, syrup from canned peaches, and juice from canned pineapple) caused complete or partial inactivation of *B. coagulans* cells [93]. The bacteriocin was also highly effective against thermophilic endospore formers in canned foods. In samples from canned corn and peas inoculated with a cocktail of two *G. stearothermophilus* strains, added enterocin AS-48 (7 μg/g) reduced viable cell counts below detection levels during storage of samples at 45 °C for 30 days [58].

Enterocin AS-48 (4–7 μg/mL) could also find application in bread and bakery products. Enterocin AS-48 was effective against proliferation of rope-forming *B. subtilis* and *B. licheniformis*, as well as *B. cereus* and *Bacillus pumilus* strains in experimental dough from wheat flour [94]. In bakery ingredients, inhibition of *S. aureus* by enterocin AS-48 (50 μg/mL) greatly depended on the food substrate, ranging from complete inactivation in liquid caramel, partial but significant inactivation in substrates like pumpkin comfiture or diluted almond cream, to non-significant inhibition (as in vanilla or chocolate creams) [95]. In desserts, highest inactivation of *S. aureus* by added bacteriocin (50 μg/mL) was observed in baker’s cream, while lowest activity was detected in yogurt-type soy-based desserts and in gelatin puddings [96]. *B. cereus*, and *L. monocytogenes* also could be controlled by bacteriocin addition (added at final concentrations of 25–50 or 15–25 μg/mL, respectively). Interestingly, addition of enterocin AS-48 to gelatin pudding prevented gelatin liquefaction caused by proteases from *Bacillus.* In general, it was observed that the bacteriocin had lower efficacy in soy-based desserts.

**Table 1:** Control of foodborne pathogenic or spoilage bacteria in different food systems by enterocin AS-48 applied singly or in combination with other hurdles.

Target Bacterium	Food Substrate	Reference(s)
*Bacillus cereus*	Cheese, rice gruel, cooked rice, sprouts, green asparagus, whole fruit pieces and sliced fruits, vegetable soups, wheat dough, desserts	[55,76,82,92,94,96]
*Bacillus weihenstephanensis*	Sprouts, green asparagus	[82]
*Bacillus licheniformis*	Apple cider, energy drinks, wheat dough	[56,91,94]
*Bacillus coagulans*	Canned foods	[93]
*Bacillus subtilis*	Wheat dough	[94]
*Paenibacillus* spp*.*	Vegetable soups	[92]
*Geobacillus stearothermophilus*	Canned foods and coconut juice	[58]
*Alicyclobacillus acidoterrestris*	Fruit juices	[57]
*Listeria monocytogenes*	Meat sausages, cooked ham, fermented sausage, skim milk, sprouts, green asparagus, whole fruit pieces and sliced fruits, ready-to-eat salads, energy drinks, desserts	[42,71,73,74,80,81,85,91,96]
*Staphylococcus aureus*	Meat sausages, cooked ham, fermented sausage, skim milk, cheese, sauces, energy drinks, bakery ingredients, desserts	[42,72,74,77,78,87,91,95,96]
*Staphylococcus carnosus*	Cooked ham	[75]
*Brochothrix thermosphacta*	Cooked ham	[75]
*Lactobacillus sakei*	Cooked ham	[75]
*Lactobacillus collinoides*	Apple cider	[88]
*Lactobacillus diolivorans*	Apple cider	[88]
*Pediococcus parvulus*	Apple cider	[88]
*Escherichia coli*	Apple juice, soybean sprouts	[50,83]
*Salmonella enterica*	Fermented sausage, soybean sprouts, ready-to-eat salads, apple juice	[52,74,83,86]
*Shigella flexneri*	Soybean sprouts	[83]
*Enterobacter aerogenes*	Soybean sprouts	[83]
*Yersinia enterocolitica*	Soybean sprouts	[83]
*Aeromonas hydrophila*	Soybean sprouts	[83]
*Pseudomonas fluorescens*	Soybean sprouts	[83]

## 6. Conclusions

Bacteria associated with food systems can be potential sources of antibacterial peptides, such as bacteriocins and peptide antibiotics. Enterocin AS-48 was first described as a peptide antibiotic considering that it could have potential applications for medical purposes because of its broad antibacterial spectrum [31]. At the present time, there is a growing interest in using bacteriocins as an alternative to conventional antibiotics for application on humans or animals [97,98]. Enterocin AS-48 serves as a model molecule on how proteins and peptides can evolve and adopt unique structures in order to achieve a higher stability and greater antibacterial activity. A few other circular bacteriocins have been described to date, some of which adopt a conformational structure that resembles that of enterocin AS-48, in spite of having different amino acid sequences. Circular bacteriocins such as enterocin AS-48 are of great interest as natural antimicrobials for food preservation, either singly or as part of hurdle technology. Notwithstanding, their antibacterial activity could also be exploited in the biomedical or veterinary fields.
